# Requirement analysis for an AI-based AR assistance system for surgical tools in the operating room: stakeholder requirements and technical perspectives

**DOI:** 10.1007/s11548-024-03193-0

**Published:** 2024-06-07

**Authors:** E. Cramer, A. B. Kucharski, J. Kreimeier, S. Andreß, S. Li, C. Walk, F. Merkl, J. Högl, P. Wucherer, P. Stefan, R. von Eisenhart-Rothe, P. Enste, D. Roth

**Affiliations:** 1 Research department - Health Economy and Quality of Life, Institute for Work and Technology of the Westfälische Hochschule Gelsenkirchen Bocholt Recklinghausen, University of Applied Sciences, Munscheidstraße 14, 45886 Gelsenkirchen, Germany; 2 HEX Lab, Department for Artificial Intelligence in Biomedical Engineering, Friedrich Alexander University, Henkestraße 91, 9105 Erlangen, Germany; 3 Musculoskeletal University Center Munich at the LMU Clinic, Campus Großhadern, Marchionistraße 15, 81377 Munich, Germany; 4 Medability GmbH, Geretsrieder Straße 10a, 81379 Munich, Germany; 5 School of Medicine and Health; School of Computation, Information, and Technology; Klinikum Rechts der Isar & Orthopedics and Sports Orthopedics, Technical University of Munich, Trogerstr. 10, 81675 Munich, Germany; 6 Human-Centered Computing and Extended Reality (HEX) Lab, Technical University of Munich, School of Medicine and Health & School of Computation, Information, and Technology, Klinikum rechts der Isar, Orthopedics and Sports Orthopedics, Technical University of Munich, Trogerstr. 10, 81675 Munich, Germany

**Keywords:** Smart operating room, AI surgery, Intelligent surgery, Biomedical engineering, Augmented Reality, Surgical instruments

## Abstract

**Purpose:**

We aim to investigate the integration of augmented reality (AR) within the context of increasingly complex surgical procedures and instrument handling toward the transition to smart operating rooms (OR). In contrast to cumbersome paper-based surgical instrument manuals still used in the OR, we wish to provide surgical staff with an AR head-mounted display that provides in-situ visualization and guidance throughout the assembly process of surgical instruments. Our requirement analysis supports the development and provides guidelines for its transfer into surgical practice.

**Methods:**

A three-phase user-centered design approach was applied with online interviews, an observational study, and a workshop with two focus groups with scrub nurses, circulating nurses, surgeons, manufacturers, clinic IT staff, and members of the sterilization department. The requirement analysis was based on key criteria for usability. The data were analyzed via structured content analysis.

**Results:**

We identified twelve main problems with the current use of paper manuals. Major issues included sterile users’ inability to directly handle non-sterile manuals, missing details, and excessive text information, potentially delaying procedure performance. Major requirements for AR-driven guidance fall into the categories of design, practicability, control, and integration into the current workflow. Additionally, further recommendations for technical development could be obtained.

**Conclusion:**

In conclusion, our insights have outlined a comprehensive spectrum of requirements that are essential for the successful implementation of an AI- and AR-driven guidance for assembling surgical instruments. The consistently appreciative evaluation by stakeholders underscores the profound potential of AR and AI technology as valuable assistance and guidance.

## Introduction

In terms of patient safety, and effectiveness of surgical procedures, the “smart operating room” is playing an increasingly important role in clinical development [1] including smart imaging, smart environments, or group-based technologies [2]. Smart environments create an intelligent environment by guiding and tracking medical care providers and providing feedback. Smart images denotes either the extraction of elements from an environment and their integration into an image or the enhancement of elements from within a scene. Group-based communication technologies should improve and facilitate communication processes [2]. Technologies like AR can be used for education and training, tele-mentoring, pre-surgical planning, image-guided surgery, or collecting data in OR [3]. A high number of inpatient procedures in hospitals are performed [4], whereby many surgical procedures are characterized by complex techniques and a high number of instruments, which are manufacturer-dependent, complex to handle, and may require patient-specific adjustments. Instrument sets, which are complex to assemble, are for example dorsal stabilization systems for the spine or modular universal tumor and revision systems*.* Despite the surgical team’s expertise, assembly, adjustment, and proper use of each instrument can become an immense challenge. The cognitive demands of surgery, including assembly and use of surgical tools should not be underestimated [5]. The high number of instruments that need to be held may negatively impact performance [6]. Within this demanding and high-pressure OR setting, every team member must perform their respective role: Scrub nurses (SN) address the task of assembling and handing over the surgical instruments to the surgeon in the correct sequence always guaranteeing sterility, having technical and non-technical skills [7]. Circulating nurses (CN) work in the non-sterile area and provide the instrumenting SN with the required information or materials. Research indicates that the utilization rate of individual surgical instruments during surgery tends to be notably low, and a growing number of instruments is associated with a reduced utilization rate and a significantly higher error rate, which means that instruments often cannot be assembled correctly [8] and require ad-hoc consultation of the manufacturers’ manuals or other expertise. The CN imparts the required information, as SN are not allowed to touch the non-sterile paper manual. Instrument assembly failures delay the surgical procedure, potentially adversely affecting the surgical outcomes, e.g., prolonged anesthesia promoting venous thromboembolism jeopardizing patient safety [9] or a higher risk of surgical site infections; and a lack of information in turn impacts the use of the instruments and, their effectiveness. An evaluation of defective surgical tools by Yasuhara et al. [10] revealed that 6.3% of the tools utilized during surgery were defective with tool part deterioration accounting for the majority of defects closely followed by incorrect use of tools as the second most prevalent cause. Against this complex background, AI support is becoming increasingly interesting in surgery [11]. There are already several endeavors and projects regarding a “smart operating room” and the integration of AI and AR in the OR [12]. For example, intelligent platforms already exist to improve surgical procedures^1^ and the approach of AR to visualize structures and information is also well known [13]. Furthermore, several publications on the use of HMD exist for example for image guidance, data display, communication and education and training [14, 15]. The need for a process integrated manual, highlighting the correct parts needed, their location, and step-by-step instructions to combine the instrument using an augmented reality head-mounted display is given [16]. Our research project fits into this context and additionally combines these approaches with object recognition [17].

Our research project addresses these challenges with an AI- and AR-driven guidance system supporting the OR team with ad-hoc and in-situ information to reduce workload and increase patient treatment quality. The system will locate and identify surgical assemblies and visualize instructions at this point and context of use to outperform paper-based manuals. We analyze stakeholders’ requirements throughout the surgical workflow but focus on SNs in orthopedics and (trauma) surgery as the most frequent use cases [14]. This paper gains insights into initial user requirements and fosters valuable stakeholder adoption in this context [18] to develop a prototype. We specifically address the following research questions:

1. Which problems occur during the use of manuals and instruments?
2. Which requirements do potential users have?
3. How could the system fit into the current work situation?

## Methods

### Augmented reality prototype

**Fig. 1:**
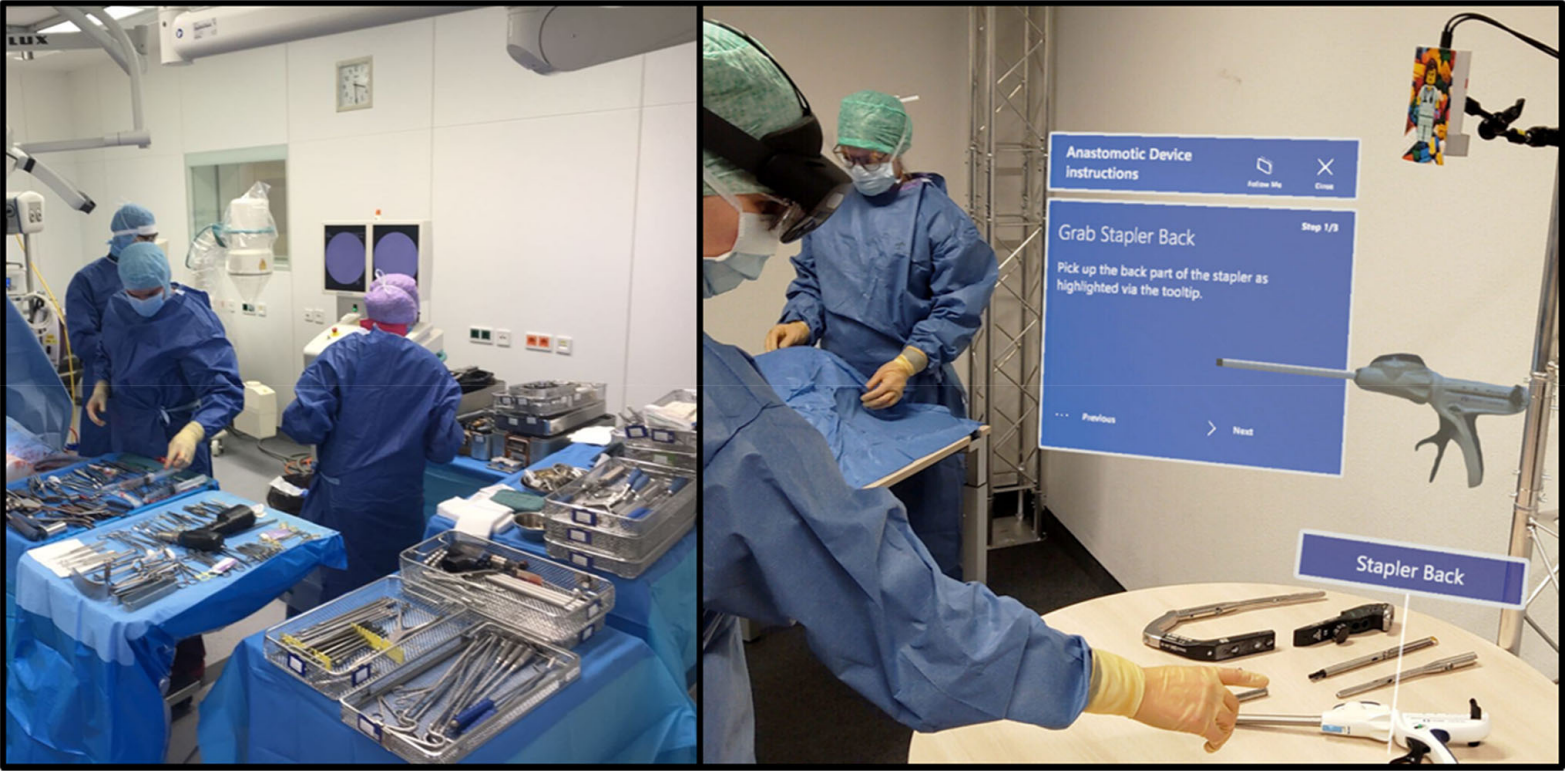
Example of the amount of surgical instruments being used during surgery (left) and photomontage concept of assembly guidance with HMD on the users head and static camera analyzing the surgical tray (right)

By superimposing content to the real world, users can perceive additional information that has no physicality. This is called AR, within the continuum between a real and virtual environment [19]. Optical-see-through devices allow for ideal stereoscopic rendering and do not block one hand to hold the device when interacting with the augmented data, e.g., using hand gestures. When combined with machine learning and computer vision approaches to estimate and track physical objects’ poses and assembly states [20], additional information can be fixed to them [21]. The augmented information has an in-situ spatial and semantic context (see Fig. 1). Such AR guidance can support the assembly processes by highlighting which parts to pick and how to assemble them. Users experience less cognitive load, make fewer mistakes, and finish the task in a shorter time [22]. However, a certain level of visual abstraction seems effective to efficiently guide users [23]. There are ambiguous insights into how different approaches of guidance visualizations’ position [22], hardware mediums [16], rendering shaders [24], and visualization dynamics [25] affect the guidance efficiency. Uni- or multimodal interaction metaphors provide benefits and limitations to point and select techniques when using augmented graphical user interfaces [26]. We use the Microsoft HoloLens 2 as a quasi-standard device for stereoscopic AR rendering, spatial sensing, and voice and gaze interaction. Moreover, we extend the HoloLens’ field-of-view-limited spatial sensing to a static depth camera (see Fig. 1). Objects that are currently not inside the HoloLens’ field-of-view can be tracked [17] and the users can be shown indicators to this specific part.

### User-centered design

We follow a user-centered design (UCD) process, which considers the needs, wants and limitations of the actual end-users during each phase of the development process [27]. The frequently cited EN ISO 9241–210 describes the guidelines of human–computer interaction and is a standard for evaluating the usability requirement [28]. Various approaches to integrating users into the development process exist, yet their effectiveness and usefulness have not been systematically compared, creating difficulty in selecting the most suitable approach from the numerous options [29]. UCD begins with an understanding of user needs and requirements [28]. Accordingly, a wide variety of methods such as gathering information, identifying user needs, visioning, assessment, and requirements specification should be included [30]. To effectively implement UCD, all project members need to become aware of future users, their work situation, their goals and tasks, how they communicate, cooperate, and interact [31].

### Research design

**Fig. 2:**
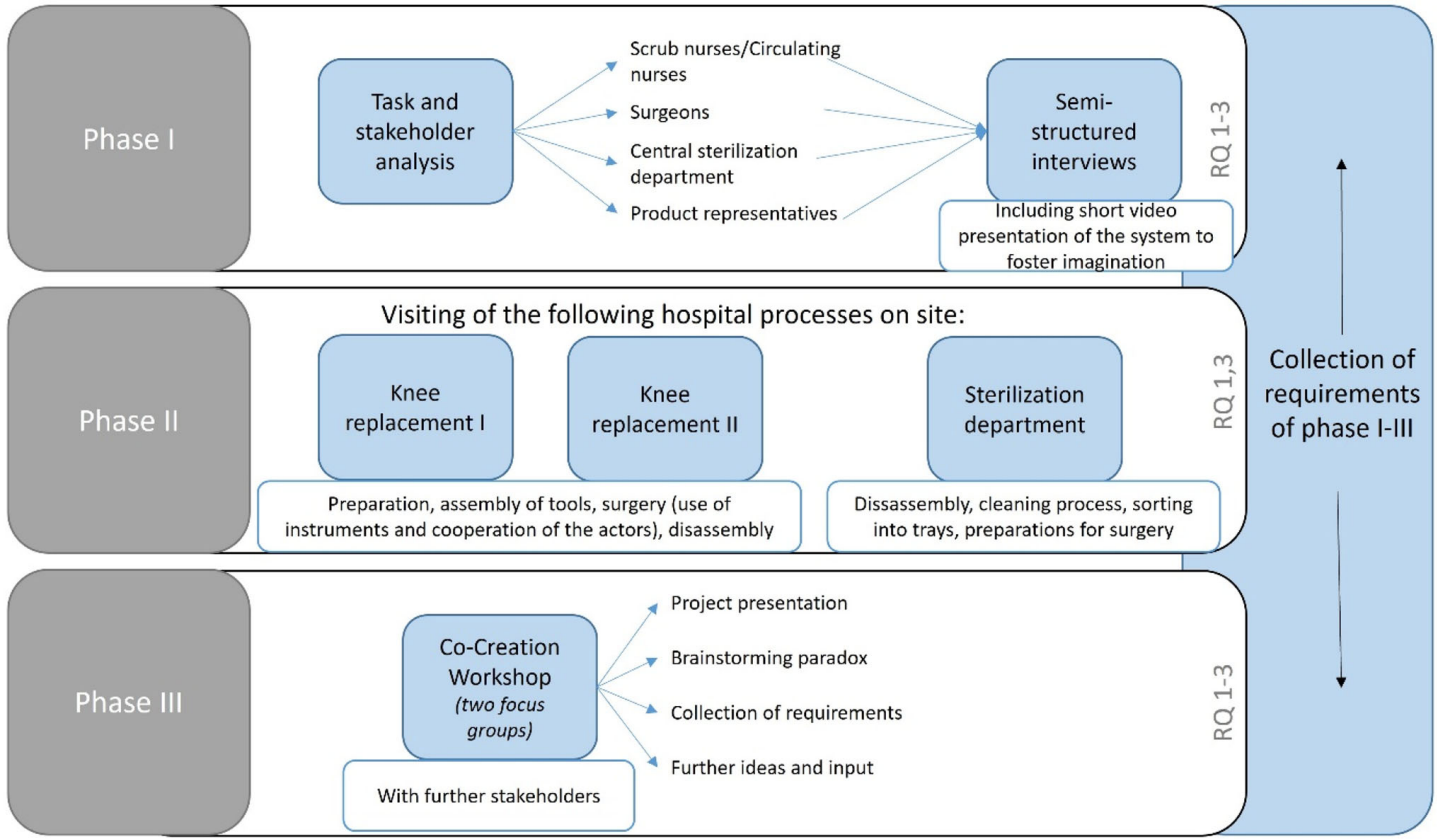
Three-phase research design. The research design consisted of a task and stakeholder analysis, observational studies and a Co-Creation Workshop. RQ = Research question

We conducted information gathering and user needs identification [30], combined with the key criteria from Gulliksen [31]. Our research project follows an iterative development process, which is beyond the scope of this article. We implemented a three-phase qualitative multi-method study design (see Fig. 2). The study received ethical approval and informed consent was obtained from all participants. In the first phase, a task and stakeholder analysis, and multiple qualitative semi-structured interviews following the recommendations of Helfferich [32] were conducted. In the second phase, the workflow of two orthopedic knee replacements and the procedures in the sterilization department were shadowed conducted by three researchers. In the third phase, a co-creation workshop was conducted with a primary focus on further requirements. It followed the basic idea of the brainstorming paradox to elicit requirements [33] and following potential problems and challenges were transformed into favorable requirements.

### Analysis strategy

All online-interviews and both focus group discussions were audio-recorded and transcribed verbatim and anonymized. Data analysis followed structured content analysis, using an inductive-deductive coding approach [34]. Two researchers independently coded all transcriptions by using QualCoder version 3.2. Coding differences were discussed until consensus was reached. The interviews and focus groups were conducted in German. The most suitable citations were translated into English for presentation in the results section.

## Results

### Participants

In phase one, six online-interviews with ten participants were conducted. In phase two, two knee replacement surgeries were observed, shadowing the workflows of all involved user groups. In phase three, 19 volunteers participated in the Co-Creation workshop and were divided into two focus groups. The work experience varied. Participant characteristics for phases one and three are provided in Table 1.

**Table 1:** Characteristics of the study population

Characteristics		*n* (%)	*n* (%)		
	Gender		Work experience		
			0–5 years	5–10 years	Above 10 years
*Interviews*					
SNs	Female	4 (40)	2 (20)	2 (20)	0 (0)
	Male	0 (0)			
Surgeons	Female	0 (0)	1 (10)	0 (0)	0 (0)
	Male	1 (10)			
Central sterilization department	Female	0 (0)	1 (10)	1 (10)	1 (10)
	Male	3 (30)			
Product representatives	Female	1 (10)	0 (0)	1 (10)	1 (10)
	Male	1 (10)			
	Male	0 (0)			
Summe		10 (100)	4 (40)	4 (40)	2 (20)
*Co creation workshop*					
SNs	Female	2 (10,5)	1 (5,3)	1 (5,3)	1 (5,3)
	Male	1 (5,3)			
Surgeons	Female	0 (0)	1 (5,3)	2 (10,5)	3 (15,8)
	Male	6 (31,6)			
Central sterilization department	Female	0 (0)	0 (0)	0 (0)	0 (0)
	Male	0 (0)			
Product representatives	Female	2 (10,5)	2 (10,5)	0 (0)	3 (15,8)
	Male	3 (15,8)			
IT department	Female	0 (0)	0 (0)	1 (5,3)	1 (5,3)
	Male	2 (10,5)			
Others (Other physicians, scientific staff)	Female	1 (5,3)	2 (10,5)	1 (5,3)	0 (0)
	Male	2 (10,5)			
Sum		19 (100)	6 (31,6)	5 (26,3)	8 (42,1)

### Current problems

**Fig. 3:**
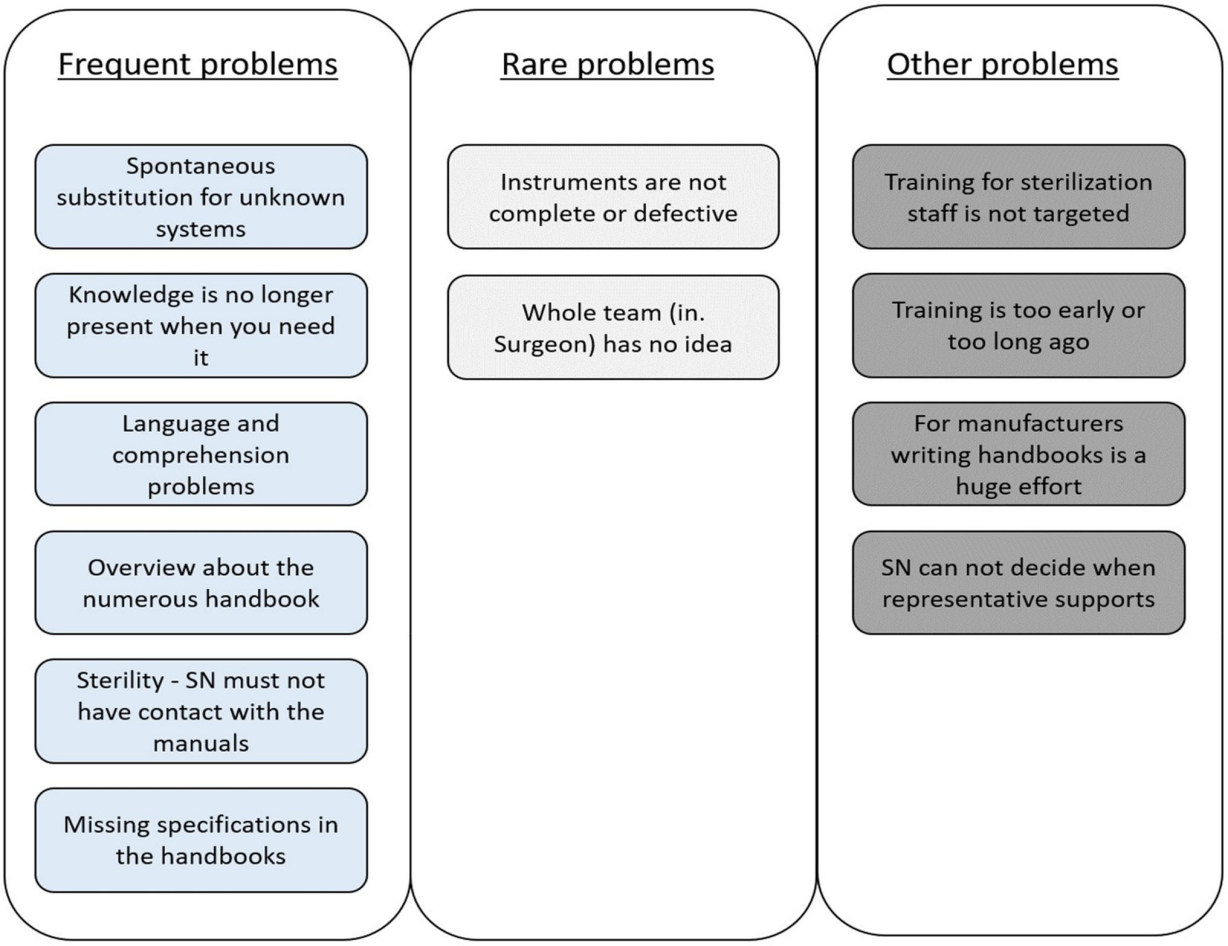
Summary of the main problems with manuals and instruments during surgery

Regarding potential problems we identified twelve main problems (see Fig. 3). Depending on the frequency they were divided into rare and frequent problems. Further problems that are not directly related to the OR situation but to manuals were summarized as other problems.

The following content provides citations and explanations for each main problem:

1. The SN is not allowed to touch the manual: *“[…] you can put the physical manuals right on a table […]. The stupid thing is that […] the jumper always has to come and you have to say, can you go back to the beginning and I*’*d like to read it again*”. *(SN)*
2. Sometimes the manuals lack or have too much text only information on specifications for the execution of minimal settings. Still, some work and setting steps are missing: *„the illustration, […] does not explain itself really“(PR*). Or „*the descriptions are a bit misleading, and that*’*s more like, that looks logical somehow, then we*’*ll do it like that*”.* (SN).*
3. If specific manuals are needed, it is “*quite annoying when you have to look for a manual first; so, we have an enormous number of instructions for operating systems and until you have found out the right one, […] it takes time […]*”.* (SN)*
4. Moreover, “*[…], there are companies that publish their manuals only in English, which is of course a bit more difficult, […]*”.* (SN).* As well, manuals „*that are written almost exclusively for surgeons, where there is absolutely nothing about the assembly of the instruments, is very difficult for us, because we see the finished instrument on the picture, as it is used, and have to deduce from this picture how we have to assemble it*”.* (SN)*.
5. Knowledge is often no longer present when you need it due to the given stressors in the situation. Even if the SNs studied the manual before, “*[…],they don*’*t have knowledge present when they need it, no matter how experienced you are now. They don*’*t have it right away*”.* (PR).*
6. When new or unfamiliar surgeries are performed, the SNs usually read the manual in advance. However, if the corresponding SN gets ill or is absent, someone else has to step in spontaneously and try to do the best possible job. This situation is very strenuous and stressful because the system is unknown.
7. From the perspective of the surgeons a serious challenge is to ensure that the right instruments are handed to them at the right time, since “*actually the big challenge [is], that everyone in the team is on the same level and so in principle the right instrument is in the right place at the right time*”. *(Surgeon)*
8. In addition, training for new instruments is often very early and “*the attention is partly very controversial*”.* (PR).* Moreover, not all employees are introduced to the handling of new instruments and “*if someone has done the training once and then months afterwards actually works with the system, then the training was useless*”* (SN).*
9. Rarely “*neither the surgeon nor we have any idea about the system, then there can be delays in the process because you*’*re not quite sure what I*’*m allowed to install*”.* (SN).*
10. The product representatives are called in only by the medical side, “*but unfortunately it*’*s not the case that we [the SNs] can choose for ourselves which surgery we want to have a representative in*”.* (SN).*
11. In the sterilization department, the trays may not be completely packed, resulting in missing instruments during surgery. Then “*[…] a sieve has to be reordered, which of course also takes time*”.* (Employee of CSD).* In addition, training for sterilization staff is not targeted and too focused on operations, which are not relevant to their workflow.
12. From the perspective of the manufacturers “*[…] writing manuals is of course also a huge effort, because we try to describe each step as precisely as possible, but also cannot go too deep, because there are simply too many different possibilities*”.* (PR).*

### Problems and fit into the current work situation

Key findings of the observation study in the OR are that the assembly of the instruments worked smoothly, before and during the observed surgery. No manuals were needed for either operation since the observed team was very experienced with the workflow of this observed surgery. No major problems with manuals and instruments were present at this specific time during the observations, but employees reported in the conversation during the observations and in the interviews that problems with manuals and instruments occur during the work processes. They lead to time delays and stressful situations. In general, an integration of an HMD would be possible, and the idea was positively evaluated by all participants.

### Guidance system requirements

The requirement analysis condensed the participants’ feedback into six categories: Design, practicability, operating and navigating within the system, system fit in the current work situation, further ideas and presumed advantages (see Table 2).

**Table 2:** Main results from the requirement analysis in terms of different requirement categories, further ideas and presumed advantages

Requirement category	Requirements	Presumed advantages of the system
Design	Clear presentation, possibility to switch off overlays, 360-degree visualization, precise visualization of different work steps, possibility for additional information, use of arrows and movements for intuitive visualization	System will add great value: supports employees who are not familiar with surgery systems uncertainties are not emphasized speed up the surgery use as training and supporting tool makes processes safer and faster
Practicability	Free decision to use system or not, supporting effect, show how instruments are assembled, easy to wear: no fogging up, combinable with eyewear, comfortable, shear on head; feedback in case of error, recognition of surgery phase, visualization of next steps in advance, quick start	
Operating and navigating within the system	Easy to handle, quick navigation to desired information, skip steps, no voice control or warning tones or hand gestures, control by gaze, visual indications, haptic cues imaginable	
System fit into the current work situation	Reliable instrument detection, precise detection of instruments and phase of surgery, users retain decision-making-autonomy, no cables, long lasting battery, easy to disinfect, enterprise WLAN capable, patient privacy, good data integration	
Further ideas	User-individual profiles, linkage between HMD of surgeon and SN, set up and adjustments at a computer, help button, live support by medical device representatives, situation-flexible navigation/ flexible AI-based adaption	

It became evident that the use of paper manuals felt outdated. There is a clear need for new solutions with the following requirements:

### Requirements for design

The system should be very clear in its presentation. It should be possible that visualizations are directly in the visual field, furthermore the visual overlays should be able to be switched off if necessary. It is wished that displayed instruments are visualized from all sides (360-degree view) “[…] because certain instruments just look the same from one side and always look completely different from the other side” (SN), so that they can be distinguished from each other. Individual work steps should be clearly visualized and if there is a need for further information, it should be possible to display this additionally. In this case text information is considered helpful but must be limited to a few words. The instructions should be visualized by arrows or movements, so that it is clear and intuitive, how the instruments should be assembled.

### Requirements for practicability

Users must be free to decide whether they want to use the system or not. The system should have a supporting effect. The system “should offer comfort, so that you don’t get a headache after wearing the glasses […] because this also affects concentration” (SN). It should be practical in its design, which means that it is easy to wear during surgery, or can be combined well with other protective eyewear, as well as not fogging up. Additionally, persons wearing glasses should be able to use the system. It should be considered that potential users with varifocals need correspondingly different visualizations. Moreover, the HMD must be stable and shear on the head, since some surgeries involve a lot of movement and the HMD must be prevented from slipping or falling off. If procedures are not correct, they should be displayed to the user or the team. It is helpful if the system could recognize the phase of the surgery and visualize the next steps for the SN in advance. It is desired that the system starts quickly.

### Requirements for operating and navigating within the system

The system must be “easy to handle and to operate and that it does not keep so many possibilities” (SN), so that a quick navigation to the desired information is possible and that some steps can be skipped. Voice control of the system and warning tones are unfavorable intraoperatively because there is a lot of talking and other sounds. The control via hand gestures also appears impractical, because there is a risk that the hands become unsterile and hands are used for other tasks. The control of the system via gaze is preferred and “visual [indications] would be pretty good”. (SN). Haptic cues (vibration at the temple) are also an option.

### Requirements that the system could fit into the current work situation

Since permanently installed displays are used to display the manuals and seem to be an alternative, they are partly permanently installed in the room and thus creating walkways. Even if other sterile portable displays could be used, the correct information must also be searched for manually in digital manuals. HMD could facilitate the navigation to the required information since our system is tracking the surgery process and can provide information at the point of need and present instructions and information accurately and at the right time. This eliminates the need to search in the manual. Most importantly, the system must have precision when detecting surgical instruments. The system “must definitely recognize all the parts on the screen, silver on silver not always easy I think”. (Surgeon). Moreover, it is very important that users retain decision-making-autonomy. If users decide to use the system only partially, it must be considered who is responsible to mount the HMD on the SNs head. Cables “would be rather annoying, […]. So, a good battery and 90 s to boot”. (SN) are preferred to prevent stumbling and work efficiently. In addition, the system must be easy to clean and disinfect. In terms of IT infrastructure, it must be WLAN capable. Also, patients’ data privacy has to be guaranteed. Necessary data must be easy to load into the system.

### Further ideas and requirements that arose during the surveys

User-individual profiles would be practical, so that the system learns who wants to see which steps and provides personalized views and information. It could be helpful if the HMD could be set up and adjusted on a computer. An automatic documentation could add value and provide additional assurance (but has been discussed controversial). An additional help button could be useful in the case that the users do not know how to proceed. Thus, a telephone or live support by PR would be promising. A situation-flexible operation should be possible, so that gaze control can be supplemented in certain situations, for example, by a foot switch. In general, it should be considered that several applications in the surgery will be based on AR in the future, so opportunities for a complementary application must be considered. The system used for tracking purposes for the sterilization department could be useful.

### Presumed advantages

It is believed that the system adds great value to employees who are not familiar with procedures and it would not “emphasize uncertainties so strongly, and on the other hand it would of course speed up the operation considerably if the SN already knows the sequence in which the instruments have to be indicated and the surgeon does not always have to say what comes next” (SN). It is believed that it can be used as a training and support tool to provide additional security to users.

## Discussion

Due to the enormous increase in manufacturer-specific surgical instruments and their complexity, digital tools to support the assembly increase in relevancy. Our results provide essential insights into the stakeholders’ requirements for an AI- and AR-driven guidance system for assembling surgical instruments at an early stage of development. Compared to previous approaches and other methods (like analysis of affecting factors [15]), the added value of this project lies in focusing on the user group of SNs, which has not yet been addressed. Moreover, a key advantage of our system lies in object recognition, whereby instruments are recognized, their position is visually highlighted and animations are used to illustrate how they are assembled. By applying the UCD, our results contribute to a better understanding of the user groups (SNs) and use cases uncovering important requirements for technological development. Toward technical challenges, important requirements can be deduced for interaction aspects and object tracking. The specific work environment requires at best gaze-driven interaction, since hand gestures cannot be performed, and speech interaction appears not viable. Therefore, specific metaphors for pointing and selecting must be considered. Animations for each assembly step and additional text information need to be as short and precise as possible for the users. In terms of machine learning, small, similar, and complex surgical instruments with metallic-shiny or bloody surfaces make object pose estimation and assembly state very challenging. These findings can specify further technical development steps for our prototype regarding the hands-free interaction and custom-fit machine-learned object tracking for spot-on visualizations. In the following project progress, the system will be tested frequently by future users to collect feedback on the current developments based on this requirement analysis and further improve and develop the system by performing user studies.

This study faces multiple limitations. The present work was based on a small and monocentric sample size. The sample is not fully representative of the relevant stakeholders and we cannot draw general conclusions since our insights are based on subjective perspectives. Still, these perspectives offered formative insights for early developmental work and are pioneering work for future technical developments in our use case.

Current surgical literature estimates AR as the standard technology of the future [35]. Involving potential users in development is essential to reveal surgical added value [30], e.g., improve patient safety and surgical procedures’ efficiency, reduce the learning curve, and ultimately lead to better patient outcomes [36]. We join this vision with the first stakeholder-specific requirements toward a prototype to reduce the error frequency and ensure time savings. Beyond medical imaging visualization [16], e.g., in urology and neurosurgery [18]. Our future work will provide a prototype that is deduced from the requirements and OR user studies to assess the surgical workflow benefits within demanding OR limitations [14]. Our results unveil stakeholders’ requirements and the surgery teams’ need and demand for on-purpose digital support. We also aim to extend the user group from SN to central sterilization department and surgeons with varying requirements, but a similar hardware framework. The central sterilization department did not use manuals during the observation study, but in the sterilization department instrument care and maintenance is also complex and extensive. A digital support capability would be helpful because precise information on instrument maintenance and care are useful for the employees.

The future smart OR will require new procedures and assistance technologies for highly specialized and technologized teams, structures, and devices. Our project contributes to this vision by providing pre- and intraoperative stakeholder support scenarios which can improve healthcare in the long term.

## Footnotes

1 Such as ExEx.AI: https://exex.ai/
